# Identification of Novel Tumor Antigens and the Immune Landscapes of Bladder Cancer Patients for mRNA Vaccine Development

**DOI:** 10.3389/fonc.2022.921711

**Published:** 2022-06-24

**Authors:** Guixin Wang, Yukui Gao, Yanzhuo Chen, Keruo Wang, Shicheng Zhang, Gang Li

**Affiliations:** Tianjin Institute of Urology, Second Hospital of Tianjin Medical University, Tianjin, China

**Keywords:** mRNA vaccine, bladder cancer, antigens, immune landscape, immune subtypes

## Abstract

**Background:**

mRNA vaccines are a novel technology that provide a potential strategy for cancer treatment. However, few studies exist that are focused on the application and development of mRNA vaccines in bladder cancer (BLCA). Therefore, this study filtered candidate antigens and specific mRNA-suitable populations in BLCA *via* comprehensive multi-omics analysis.

**Methods:**

Clinical information, follow-up information, and gene expression profiles were obtained from the TCGA and GEO databases. Somatic mutation and DNA copy number variation of BLCA were visualized by cBioPortal. Significant survival genes were analyzed by GEPIA2. TIMER was used to evaluate the connection between candidate antigens and infiltration of antigen-presenting cells. Consensus clustering analysis was performed to identify immune subtypes using the ConsensusClusterPlus package. The Monocle package was used to visualize the immune landscapes of each BLCA patient. Weighted gene co-expression network analysis (WGCNA) was used to identify key genes for mRNA vaccines.

**Results:**

*AP2S1*, *P3H4*, and *RAC3* were identified as candidate tumor-specific antigens for BLCA. Three immune subtypes were classified based on immune-related gene expression profiles. Patients with the BCS2 subtype were characterized as immune “cold” and exhibited upregulation of immunogenic cell death modulators, whereas patients with BCS1 and BCS3 were immune “hot” and had upregulation of immune checkpoints. Interestingly, patients with the BCS2 subtype had a better prognosis than other subtypes. The immune landscapes of each patient were visualized and revealed the heterogeneity within the BCS1 subtype. Finally, 13 key immune genes were identified.

**Conclusions:**

*AP2S1*, *P3H4*, and *RAC3* were identified as candidate tumor-specific antigens, and patients with the BCS2 and BCS1A subtypes were identified as candidate populations for mRNA vaccines. In summary, this study provides novel insights and a theoretical basis for mRNA vaccine development in BLCA and other malignancies.

## Introduction

Bladder cancer (BLCA) has one of the highest mortality rates of all cancers and has been estimated to cause more than 170,000 deaths in the world annually (1, 2). The treatment for bladder cancer is dependent on diagnosis *via* transurethral resection of bladder tumor (TURBT). For non-muscle invasive bladder cancer (NMIBC), Bacille Calmette-Guerin (BCG) treatment is the standard immunotherapy for non-high risk NMIBC patients (3). However, most NMIBC patients will experience recurrence within 5 years, and a portion of patients will develop muscle-invasive bladder cancer (MIBC). MIBC is an aggressive disease associated with high morbidity and mortality, and life prolonging therapies include radical cystectomy, chemotherapy, radiotherapy, and immune checkpoint inhibitors (4). Nevertheless, the 5-year survival rate for nonmetastatic MIBC patients (75% of newly diagnosed bladder cancer) is only 36% - 48% after first-line treatment, while the 5-year relative survival is 5% - 36% in metastatic MIBC (3). Therefore, novel treatments are needed to improve the prognosis of BLCA patients.

Owing to the worldwide spread of COVID-19, mRNA vaccines have received increasing interest (5, 6). Although research on traditional cancer vaccines has been conducted for many years, the application of cancer vaccines is still limited (7). At present, only a few areas of mRNA vaccine research have made progress, such as prostate cancer, non-small cell lung cancer, melanoma and so on (8–10). As an emerging vaccine technology, mRNA vaccines represent a promising alternative to conventional vaccine approaches due to their high potency, capacity for rapid development, and potential for low-cost manufacturing and safe administration (11). Recent years, the shortages of mRNA vaccines have been improved by new delivery strategies and materials (12). Moreover, the rise of high-throughput sequencing has spawned an increasing number of studies on personalized cancer vaccines (13). Therefore, the development and application of mRNA vaccines is feasible and urgently needed to improve the prognosis of BLCA patients.

To our knowledge, there is limited research on anti-BLCA mRNA vaccine development. It remains a challenge for oncologists to filter tumor-specific antigens for BLCA. Owing to the heterogeneity (age, tumor mutation burden, and tumor stage) of each patient, the tumor microenvironment also varies (14). The mRNA vaccine can enhance T cell response by antigen stimulation and APCs presentation, resulting in the transformation of cold tumor into hot tumor (15). Therefore, a portion of BLCA patients cannot benefit from mRNA vaccines. Given these factors, identification of specific populations suitable for mRNA vaccines by traditional methods may not be an effective strategy. In comparison, construction of the immune landscape of BLCA based on immune gene expression in BLCA patients is a potential strategy for mRNA vaccine development.

In this study, we identified tumor-specific antigens and a specific patient population suitable for anti-BLCA mRNA vaccine development. We identified three potential tumor-specific antigens in BLCA by integrating multiple omics data, and found that the immune subtype population with “cold” tumors adapted to the mRNA vaccine. Further, 13 key immune-related genes were identified for mRNA vaccine development *via* weighted gene co-expression network analysis (WGCNA). Taken together, our findings provide novel insights into the development of anti-BLCA mRNA vaccines.

## Materials and Methods

### Data Collection and Preprocessing

The gene expression profiles and clinical information of 412 primary bladder cancer patients were downloaded from The Cancer Genome Atlas (TCGA) using the TCGAbiolinks package. The GSE13507 dataset, which was downloaded from the GEO database (https://www.ncbi.nlm.nih.gov/gds/?term=GSE13507), includes the gene expression profiles and follow-up information of 165 primary bladder cancer patients. GSE13507 was used as the training dataset, while the TCGA-BLCA dataset was used as the validation dataset. A total of 1,793 immune-related genes and immune-related signatures were obtained from the IMMPORT database (https://immport.niaid.nih.gov/home).

GPL6102 probes were used to map the GSE13507 dataset, and gene expression value was normalized for subsequent analysis. TCGA-BLCA expression was transformed into transcripts per kilobase of exon model per million mapped reads (TPM). Patients without complete follow-up information were excluded, and finally 406 TCGA-BLCA patients were included in the analysis.

### cBioPortal Analysis

The cBioPortal database (https://www.cbioportal.org/) was utilized to analyze DNA copy number variation (CNV) and single nucleotide variation (SNV) of the TCGA dataset (16). The CNV landscapes and gene alternations of BLCA were analyzed.

### GEPIA2 and Survival Analysis

The GEPIA2 database (http://gepia2.cancer-pku.cn/#index) is a platform that integrates the RNA-seq data and follow-up information of multiple cancers (17). The top 500 most significant overall survival and disease-free survival genes were downloaded from the GEPIA2 database. The group cutoff was set as a median value. *P*-values < 0.05 were considered statistically significant. The Kaplan-Meier curves of seven candidate genes were obtained.

### TIMER Analysis

The TIMER database (https://cistrome.shinyapps.io/timer/) was used to explore the association between the abundance of three immune cell types (B cells, macrophages, and dendritic cells) and candidate target genes (18). The correlation between gene expression and the abundance of immune cells was adjusted by tumor purity using Spearman’s correlation analysis. *P*-values < 0.05 were considered significant.

### Identification and Validation of Immune Subtypes

The 1,793 immune-related genes were analyzed *via* univariate Cox hazard analysis in GSE13507, and the 52 most significant immune-related survival genes with *P*-values < 0.01 were obtained. Subsequently, a consistency matrix was built to identify the immune-related subtypes using the ConsensusClusterPlus package. The PAM algorithm was applied and 100 bootstraps were performed, each involving 80% of the patients in the discovery cohort. Cluster sets varied from two to six. The immune subtypes were also validated in TCGA dataset in the same manner. The clinical characteristics of different immune subtypes were explored.

The SNV landscapes and tumor mutation burden (TMB) of each patient were analyzed using the maftools package. The TMB differences between immune subtypes were tested by Kruskal-Wallis test. P-values < 0.05 were considered statistically significant.

### Prognostic and Immune-Infiltration Evaluation of Immune Subtypes

Survival analysis was performed for patients with overall survival data in the different immune subtypes using the Kaplan-Meier method; *P* values < 0.05 were considered statistically significant. The immune-pathway signatures and 28 immune cell signatures were collected from public research (19). The single-sample GSEA (ssGSEA) was used to calculate the enrichment score of each sample. Kruskal-Wallis test was used to estimate the differences between immune subtypes. Six immune subtypes of BLCA were evaluated using the ImmuneSubtypeClassifier package (20).

### Identification of Immune Landscapes

The GSE13507 dataset was used for dimensionality reduction analysis using the monocle package to visualize the distribution of each patient. In addition, the 52 most significant survival genes were set as cluster genes. The maximum number of components was set to 2, and the discriminative dimensionality reduction with trees was used. Finally, the PCA1 and PCA2 of the immune landscape were extracted to perform a correlation analysis with 28 immune cells *via* Pearson correlation analysis. P-values < 0.05 were considered statistically significant. Survival analysis was performed on four distinct state subtypes.

### WGCNA

A total of 165 patients in the GSE13507 dataset were included in the WGCNA, and seven gene modules were obtained. Univariate Cox regression analysis was conducted to estimate the prognostic value of different gene modules. Gene ontology and KEGG analyses were annotated using the clusterProfiler package. Pearson correlation analysis was used to evaluate the relationship between PCA1 in the immune landscape and red gene module. Finally, the Metascape database (https://metascape.org/gp/index.html#/main/step1) was used to identify the hub genes of the red gene module (21).

## Results

### Identification of Candidate Neoantigens in BLCA

**Figure 1:**
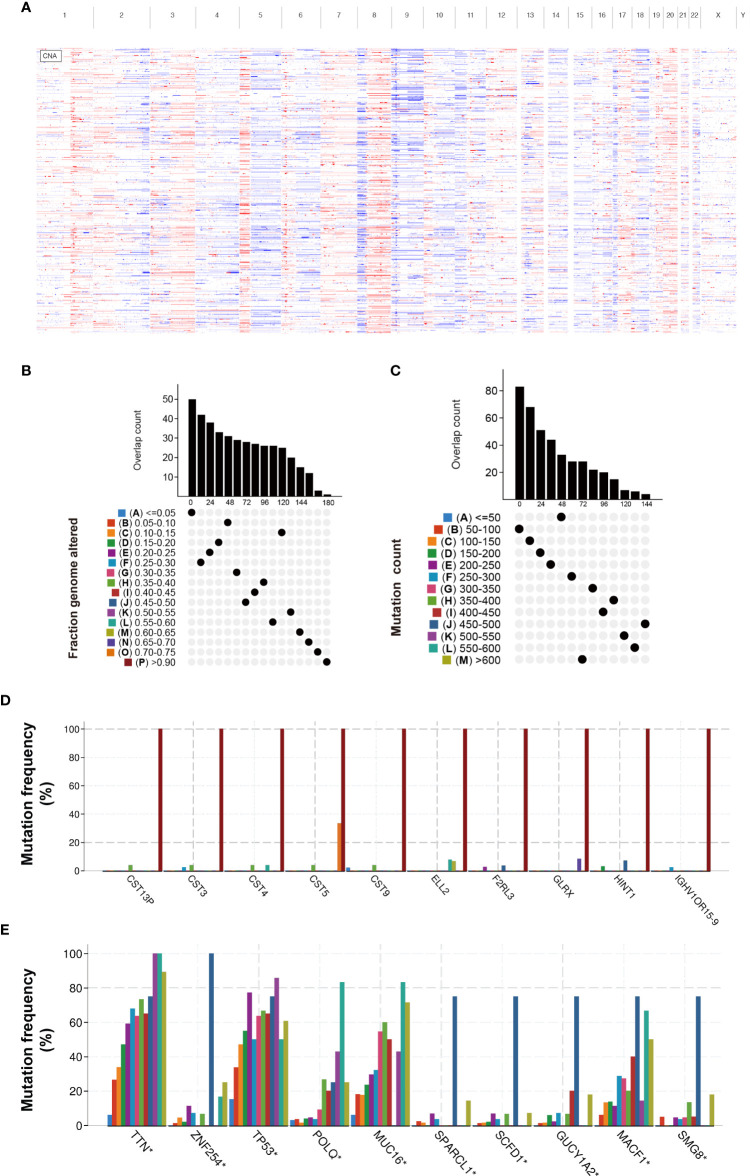
Identification of candidate neoantigens in BLCA. **(A)** Chromosomal distribution of amplified and deleted copy number genes in BLCA. **(B)** Overlapping mutated genes distributed in the distinct genome alteration group. **(C)** Overlapping mutated genes distributed in the distinct mutation count group. **(D)** The genes with the highest frequency in the genome alteration group. **(E)** The genes with the highest frequency in the mutation count group. *P< 0.05.

To screen candidate tumor-specific antigens in BLCA for mRNA vaccine development, we first comprehensively analyzed genome alterations (SNV and CNV) in the TCGA-BLCA dataset (**Figure 1A**). As shown in **Figures 1B, C**, a total of 16,926 mutated genes which potentially encode tumor-specific antigens were selected by evaluating mutation counts and altered genome fractions in each patient. Genome alterations analysis identified the top 10 genes with the highest frequency of mutational counts and altered genome fractions (**Figures 1D, E**). Notably, Titin (*TTN*), Tumor Protein P53 (*TP53*), and Cancer Antigen 125 (*MUC16*) have been confirmed to play a critical role in tumor development in multiple cancers. Interestingly, we found that the cystatin (CST) superfamily members had the highest altered genome fraction in BLCA, which suggests potential target antigens for mRNA vaccines. Taken together, these data identified many candidate mutated genes for mRNA vaccine development.

### Identification of Tumor-Specific Antigens Related to the Prognosis of BLCA

**Figure 2:**
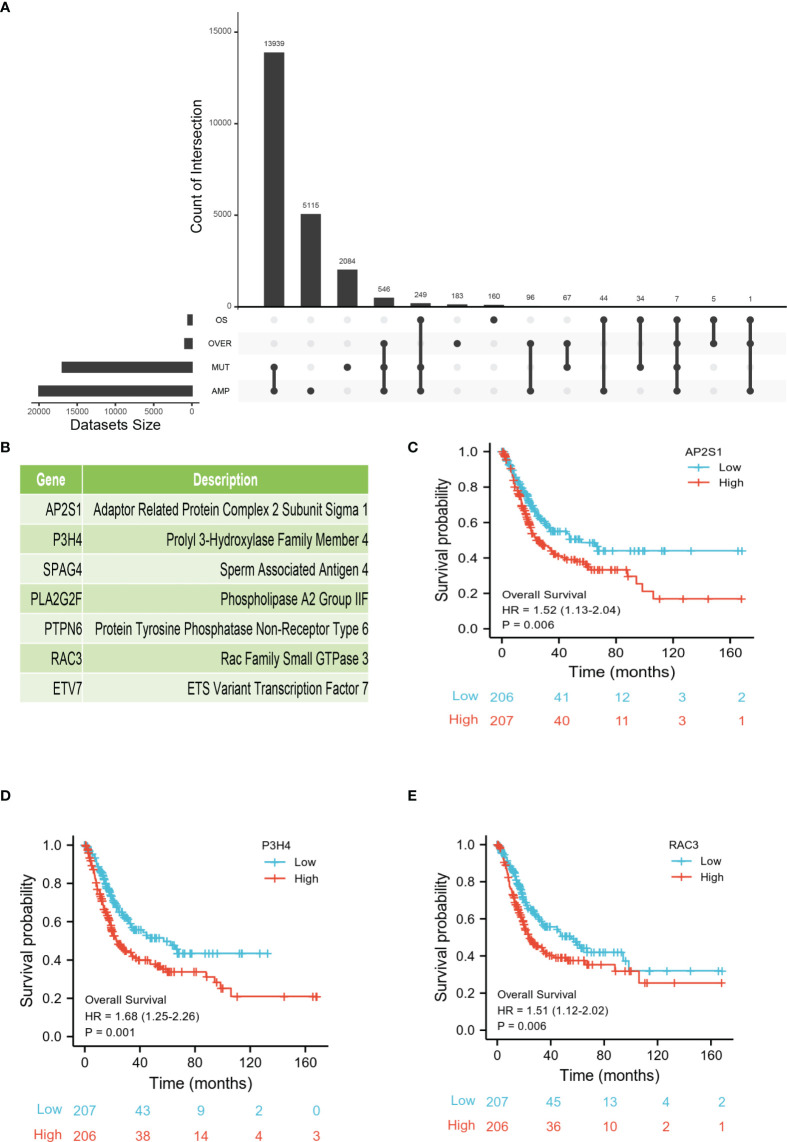
Identification of tumor-specific antigens related to prognosis of BLCA. **(A)** Upset diagram shows the number of gene intersections in different groups. **(B)** Seven candidate protein-coding genes. **(C–E)** Kaplan-Meier OS of *AP2S1* **(C)**, *P3H4* **(D)**, and *RAC3* **(E)** in BLCA.

To further explore the most valuable tumor-specific antigens, we collected OS- and DFS-related genes in BLCA. After integrating four gene sets including mutational genes, amplification genes, OS-related genes, and DFS-related genes, a total of seven genes were identified (**Figure 2A**). As shown in **Figure 2B**, *AP2S1*, *P3H4* (*SC65*), *SPAG4*, *PLA2G2F*, *PTPN6*, *RAC3*, and *ETV7* were identified as candidate tumor-associated antigens. Subsequently, the Kaplan-Meier survival curves of the seven genes were analyzed using the GEPIA2 database. Higher expression levels of *AP2S1*, *P3H4* (*SC65*), and *RAC3* were associated with poor prognosis, suggesting the potential immune-stimulatory effect of these genes (**Figures 2C–E**). However, the other genes (*SPAG4*, *PLA2G2F*, *PTPN6*, and *ETV7*) were associated with a better prognosis (**Figures S1A–D**). In total, three potential tumor-specific antigens (*AP2S1*, *P3H4*, *RAC3*) were identified for mRNA vaccine research.

### Correlation Between Candidate Tumor Antigens and Antigen-Presenting Cells

**Figure 3:**
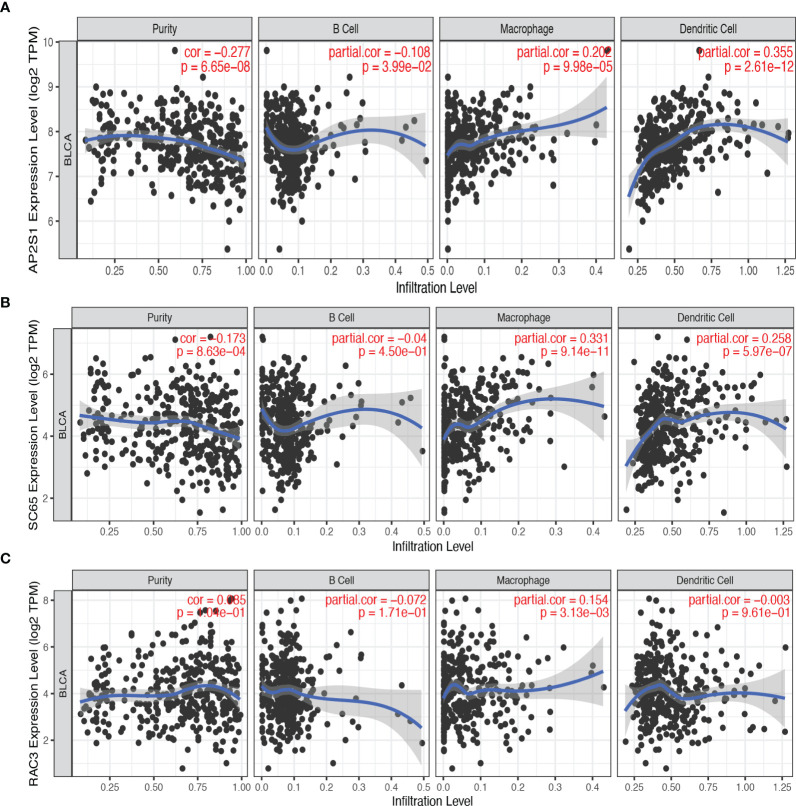
The correlation between candidate tumor antigens and antigen-presenting cells. **(A)** Association of *AP2S1* expression with the purity of infiltrating cells and the number of macrophages, dendritic cells, and B cells in BLCA. **(B)** Association of *P3H4* (*SC65*) expression with the purity of infiltrating cells and the number of macrophages, dendritic cells, and B cells in BLCA. **(C)** Association of *RAC3* expression with the purity of infiltrating cells and the number of macrophages, dendritic cells, and B cells in BLCA.

Antigen-presenting cells (APCs) include B cells, dendritic cells, and macrophages. These cells play a critical role in antigen recognition and the induction of immune responses (22). Therefore, we explored the relationship between three candidate genes and APC. As shown in **Figures 3A–C**, the expression of *AP2S1* was positively correlated with the abundance of macrophages (*P* < 0.001) and dendritic cells (*P* < 0.001). High expression of *P3H4* (*SC65*) was also associated with the abundance of macrophages (*P* < 0.001) and dendritic cells (*P* < 0.001). Additionally, the expression of *RAC3* was positively related to the level of macrophages (*P* < 0.001). These results implied that *AP2S1*, *P3H4*, and *RAC3* may be processed by APC for the induction of immune responses.

### Identification and Validation of Novel Immune Subtypes of BLCA

**Figure 4:**
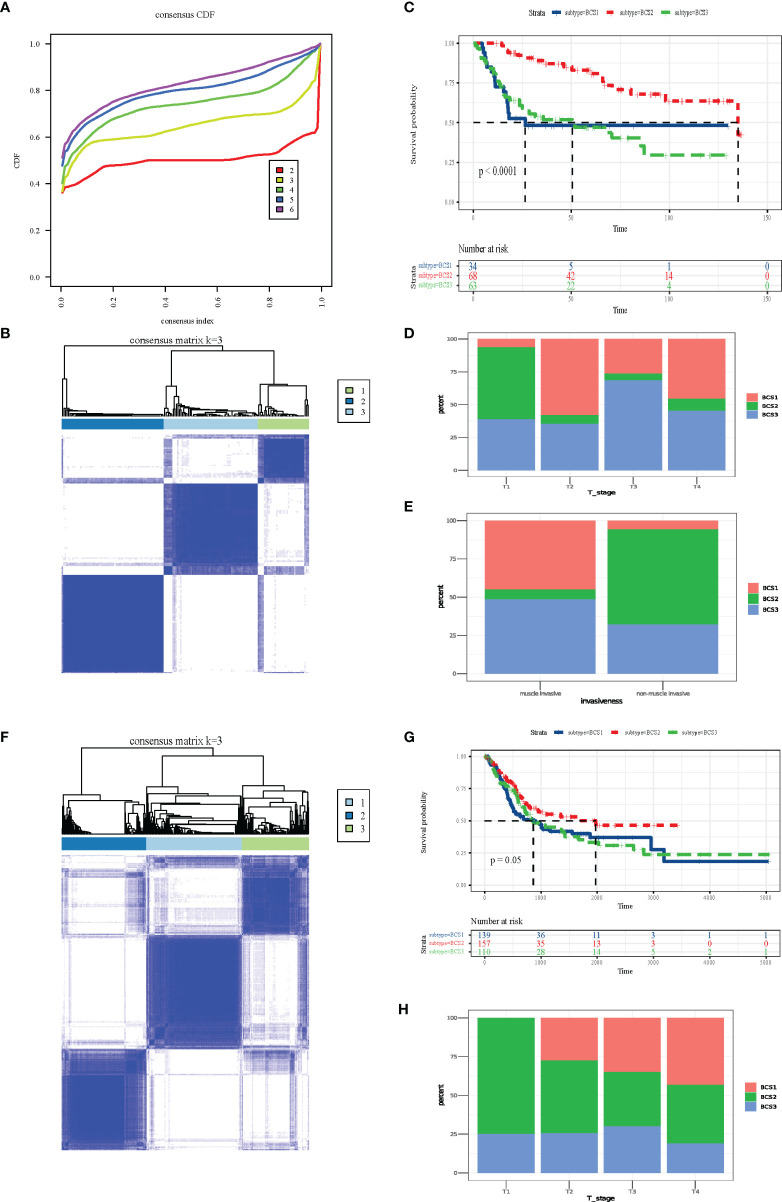
Identification and validation of novel immune subtypes of BLCA. **(A)** Cumulative distribution function curve of immune-related genes in the GEO cohort. **(B)** Sample clustering heat map in the GEO cohort. **(C)** Kaplan-Meier curves showing OS of BLCA immune subtypes in the GEO cohort. **(D, E)** Distribution of BCS1-BCS3 across BLCA **(D)** T-stage and **(E)** invasiveness in the GEO cohort. **(F)** Sample clustering heat map in the TCGA cohort. **(G)** Kaplan-Meier curves showing OS of the BLCA immune subtypes in the TCGA cohort. **(H)** Distribution of BCS1-BCS3 across BLCA T-stage in the TCGA cohort.

The heterogeneity of the tumor immune microenvironment results in distinct outcomes in response to immunotherapy and mRNA vaccines (15). To identify suitable immune subtypes for mRNA vaccines, a total of 1,793 immune-related genes in the GSE13507 dataset were analyzed *via* univariate Cox regression analysis, and the 52 most significant survival genes (*P* < 0.01) were subsequently included in the cluster analysis (**Table S1**). The clinical characteristics of the GEO and TCGA cohorts are shown in **Table S2**. As shown in **Figure 4A**, we chose the classification of three. The cluster heatmap shows the results of the cluster analysis (**Figure 4B**). The survival analysis suggested that three subtypes (BCS1, BCS2, and BCS3) exhibited completely different prognoses (*P* < 0.05, **Figure 4C**). Stacking charts show that the BCS2 subtype was associated with pathologic T1 stage and non-muscle invasive status (**Figures 4D, E**). Subsequently, we performed the same clustering method on the TCGA dataset (**Figure 4F**). Consistently, BCS2 subtype patients had a better prognosis (*P* = 0.05, **Figure 4G**). In addition, the BCS2 subtype was correlated with T1 stage (**Figure 4H**). Together, these data show that we identified and validated three immune-related subtypes in distinct datasets, and the BCS1 subtype was associated with poor survival, while the BCS2 subtype was associated with a better prognosis. Therefore, novel immune subtype identification could be used a prognostic biomarker in BLCA patients.

### Connection Between Immune Subtypes and the Tumor Mutational Landscape

**Figure 5:**
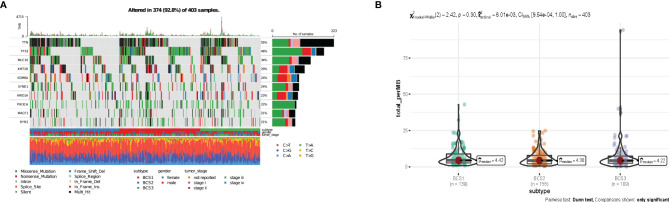
The connection between immune subtyipes and the tumor mutational landscape. **(A)** Ten highly mutated genes in the BLCA immune subtypes. **(B)** TMB in BLCA BCS1-BCS3.

It has been reported that tumor mutation burden (TMB) is associated with response to immunotherapy, including mRNA vaccine effects (23, 24). To characterize the genome heterogeneity of the three immune subtypes, mutect2-processed data from 403 patients from the TCGA-BLCA database was evaluated using the maftools package. However, no significant variation was observed in the three immune subtypes (**Figure 5A**). Also, there was no significant difference in tumor mutation burden in the three immune subtypes (*P* = 0.30, **Figure 5B**). In summary, TMB may not be an accurate indicator for predicting immune response to mRNA vaccines, as the immune subtypes we constructed showed no significant difference.

### Association Between Anti-Tumor Immunity Between the Three Immune Subtypes

**Figure 6:**
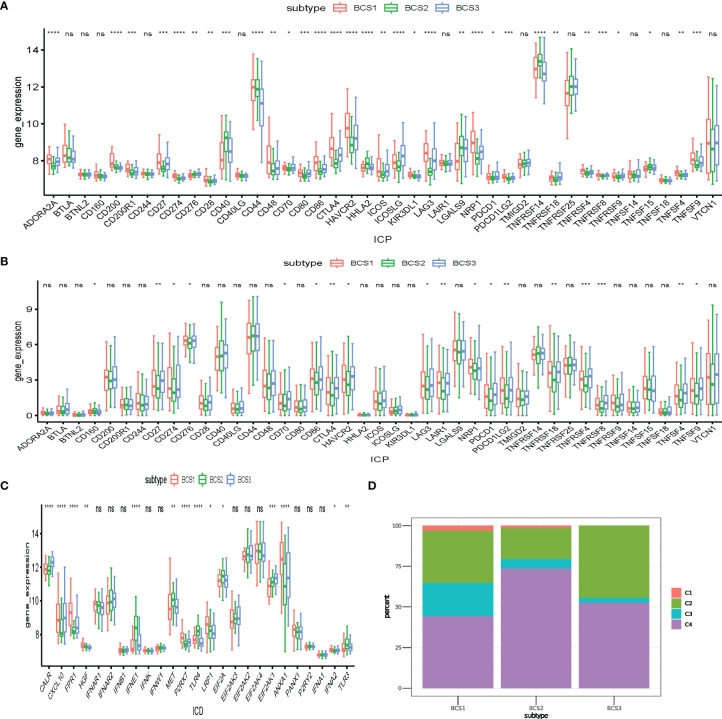
Association between anti-tumor immunity between the three immune subtypes. **(A, B)** Differential expression of ICP genes among the BLCA immune subtypes in the **(A)** GEO and **(B)** TCGA cohorts. **(C)** Differential expression of ICD modulator genes among the BLCA immune subtypes in the GEO cohort. **(D)** Overlap of BLCA immune subtypes with 6 pan-cancer immune subtypes. *P < 0.05, **P < 0.01, ***P<0.001, ****P<0.0001, and ‘ns’ represents not significant.

Immune check points (ICP) and immunogenic cell death (ICD) regulators play essential roles in anti-tumor immunity, and may influence the efficacy of immunotherapy and mRNA vaccines (25). To determine the differences between distinct immune subtypes, we comprehensively accessed 43 ICP genes and 25 ICD regulator genes in the GSE13507 and TCGA datasets. In the GSE13507 dataset, most ICP genes (LAG3, CD274, ADORA2A) had higher expression in the BCS1 and BCS3 subtypes (**Figure 6A**). Consistently, the same result was shown in the TCGA dataset (**Figure 6B**). Additionally, patients with BCS2 exhibited a higher expression of ICD genes such as *IFNE1*, *MET*, *TLR4*, *EIF2A*, and *TLR3* in the GSE13507 dataset (**Figure 6C**). To confirm the stability of the immune subtypes we constructed, we compared the immune subtypes with the former immune cluster (**Figure 6D**). Based on a previous study (21), we found that BCS2 was associated with C4 (lymphocyte depleted), and the BCS1&3 subtype were associated with C2 (IFN-γ dominant). In other words, BCS1 and BCS3 had high expression of ICP genes, and may therefore benefit from immunotherapy. BCS2 patients with high expression of ICD genes may also be suitable for mRNA vaccines. Of note, the BCS2 subtype was associated with the lymphocyte depleted type, indicating that the C4 type may be suitable for mRNA vaccines. In summary, patients with BCS2 subtype seemed to be more suitable for vaccination.

### Immune Cell Infiltration of Distinct Immune Subtypes

**Figure 7:**
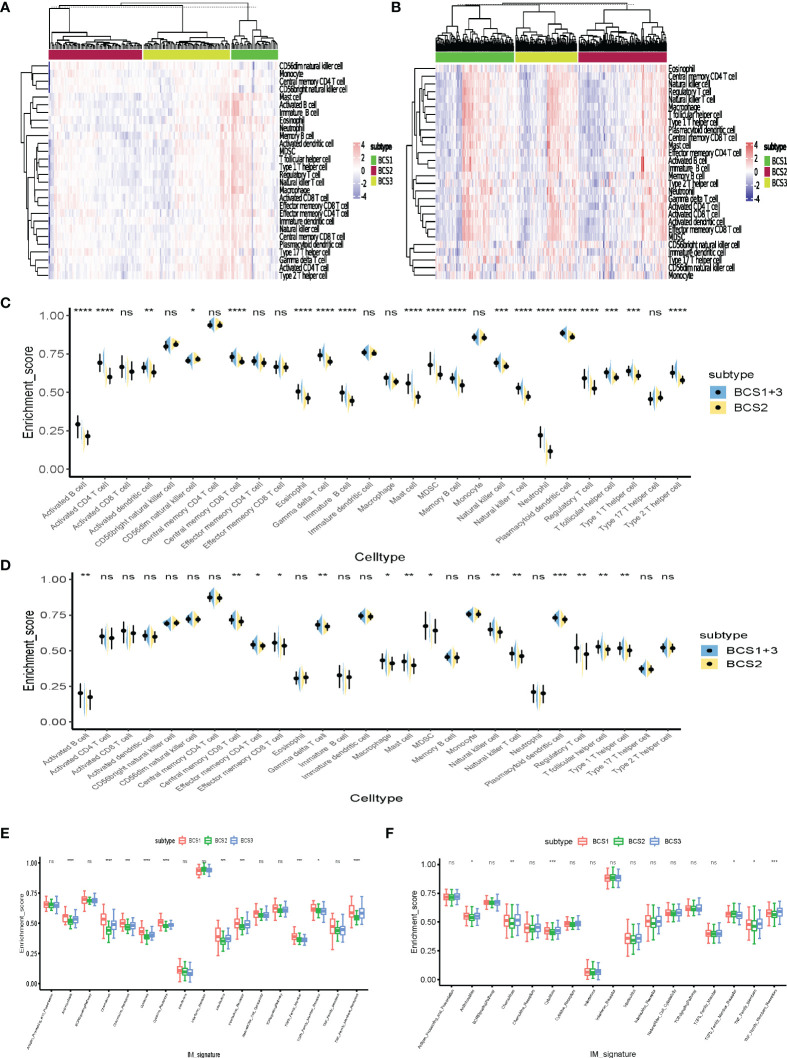
Immune cell infiltration of distinct immune subtypes. **(A)** Differential enrichment scores of 28 immune cell signatures among the BLCA immune subtypes in the GEO cohort. **(B)** Differential enrichment scores of 28 immune cell signatures among the BLCA immune subtypes in the TCGA cohort. **(C, D)** Differential enrichment scores of 28 immune cell signatures between BCS2 and other types in the GEO **(C)** and TCGA cohorts **(D)**. **(E, F)** Differential enrichment scores of 17 immune-related signature pathways among BLCA immune subtypes in the GEO **(E)** and TCGA **(F)** cohorts. *P < 0.05, **P < 0.01, ***P <0.001, ****P <0.0001, and ‘ns’ represents not significant.

Previous studies confirmed different outcomes of immunotherapy with “hot” and “cold” tumors (25). “Cold” tumors lack immune cell infiltration. Therefore, these may benefit from antigen-stimulation by mRNA vaccines to remodel the immune microenvironment. Twenty-eight tumor-infiltrating lymphocyte markers were collected and ssGSEA algorithm utilized to determine the enrichment scores of each immune cell in individual samples. The heatmap shows that BCS2 was a “cold” tumor subtype, while BCS1 and BCS3 were not “cold” tumors in the GSE13507 dataset (**Figure 7A**). Then, we validated the result in the TCGA dataset (**Figure 7B**). To better visualize the immune infiltration results, violin diagrams were constructed and showed that BCS2 had lower immune filtration in the GSE13507 and TCGA datasets (**Figures 7C, D**). Immune-related signature pathway analysis showed that antimicrobials, cytokines, and TNF-family member receptors were upregulated in the BCS1 and BCS3 subtypes in both datasets (**Figures 7E, F**). Taken together, these findings indicated that the BCS2 subtype with lower immune infiltration was a “cold” tumor suitable for mRNA vaccines, while the BCS1 and BCS3 subtypes with high expression of ICP may benefit from immunotherapy.

### Construction of the Immune Landscape of BLCA

**Figure 8:**
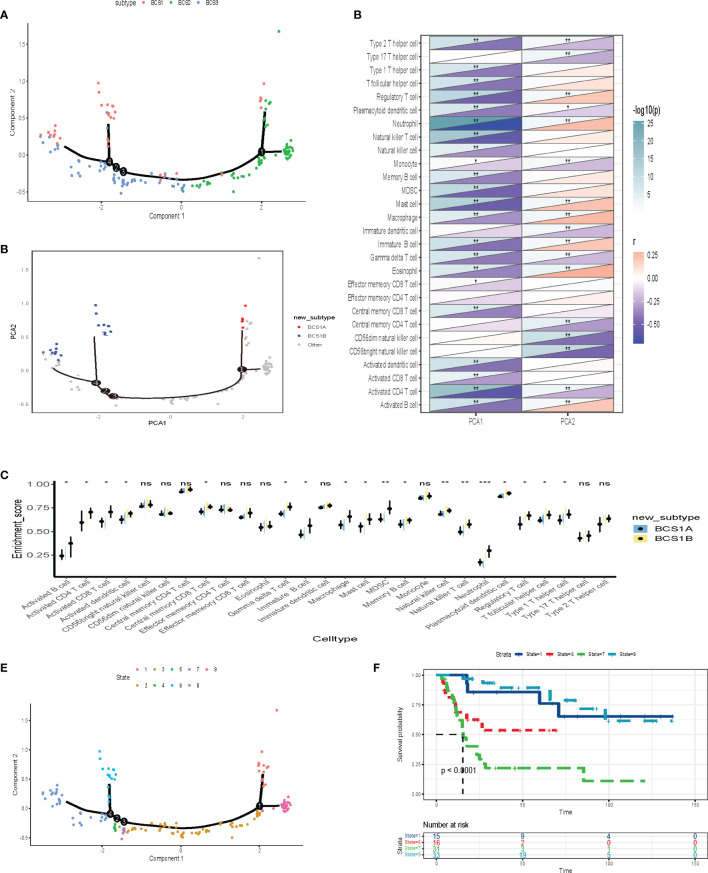
Construction of the immune landscape for BLCA. **(A)** Each point represents a patient, the immune subtypes are color-coded. The horizontal axis represents the first principal component, and the vertical axis represents the second principal component. **(B)** Immune landscape of the subsets of BLCA immune subtypes. **(C)** Heat map of two principal components with 28 immune cell signatures. **(D)** Differential enrichment scores of 28 immune cell signatures in BCS1A and BCS1B. **(E, F)** Immune landscape of samples from four extreme locations and **(F)** their prognostic status. *P < 0.05, **P < 0.01, ***P <0.001, and ‘ns’ represents not significant.

To better visualize the immune landscape of individual patients, the gene profiles of each patient in the GSE13507 dataset were used to construct immune landscapes using the monocle package. Patients with distinct immune subtypes were clearly separated in the landscape (**Figure 8A**). However, we discovered that BCS1 could be further subdivided into BCS1A and BCS1B (**Figure 8B**), and that BCS1A had lower enrichment of immune cells (**Figure 8C**). These results suggested that the immune landscape could distinguish between immune subtypes, and that BCS1A patients may be more suitable for mRNA vaccines than BCS1B patients.

Further dimensionality reduction analysis of component 1and component 2 from the immune landscape was conducted. The X-axis was negatively correlated with immune cells including Type 2 T helper cells, Type 1 T helper cells, and T follicular helper cells (**Figure 8D**). The Y-axis was positively correlated with most immune cells. To explore the prognostic effect of the immune landscape, the patients were divided into nine groups (**Figure 8E**). The survival analysis showed that four patients were clearly separated from the rest of the distribution (*P* < 0.0001, **Figure 8F**). Patients who were close to BCS2 in their landscape had a better prognosis. In summary, the immune landscape we constructed identified populations suitable for mRNA vaccines and evaluated their prognosis.

### Identification of Critical Immune-Related Gene Modules

**Figure 9:**
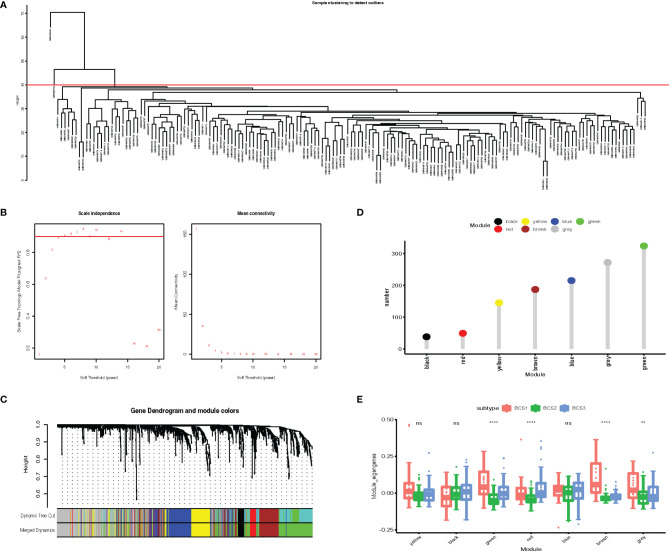
Identification of critical immune-related gene modules. **(A)** Sample clustering. **(B)** Scale-free fit index for various soft-thresholding powers. Mean connectivity for various soft-thresholding powers **(C)** Dendrogram of all differentially expressed genes clustered based on dissimilarity measure (1-TOM). **(D)** Gene numbers in each module. **(E)** Differential distribution of feature vectors of each module in the BLCA subtypes. **P < 0.01, ****P <0.0001, and ‘ns’ represents not significant.

Identification of critical immune-related genes could aid oncologists in determining whether a patient is suitable for mRNA vaccination. To identify key genes, we constructed a weighted correlation network analysis (WGCNA) based on 1,793 immune-related genes. In total, 163 samples were included in the subsequent analysis and two samples were excluded (**Figure 9A**). The soft threshold was set at five in the scale-free network (**Figure 9B**). The gene matrix was transformed into the adjacency matrix and next to the topological matrix. A minimum of 30 genes was set in each gene module. The eigengenes of each module were computed, and close modules were integrated into a new one. As shown in **Figure 9C**, seven gene modules were obtained by WGCNA analysis. The numbers of eigengenes of each module are exhibited in **Figure 9D**. The module eigengenes of BCS2 were significantly lower in the green, red, brown, and grey modules (**Figure 9E**).

### Identification of Hub Genes for BLCA mRNA Vaccine Development

**Figure 10:**
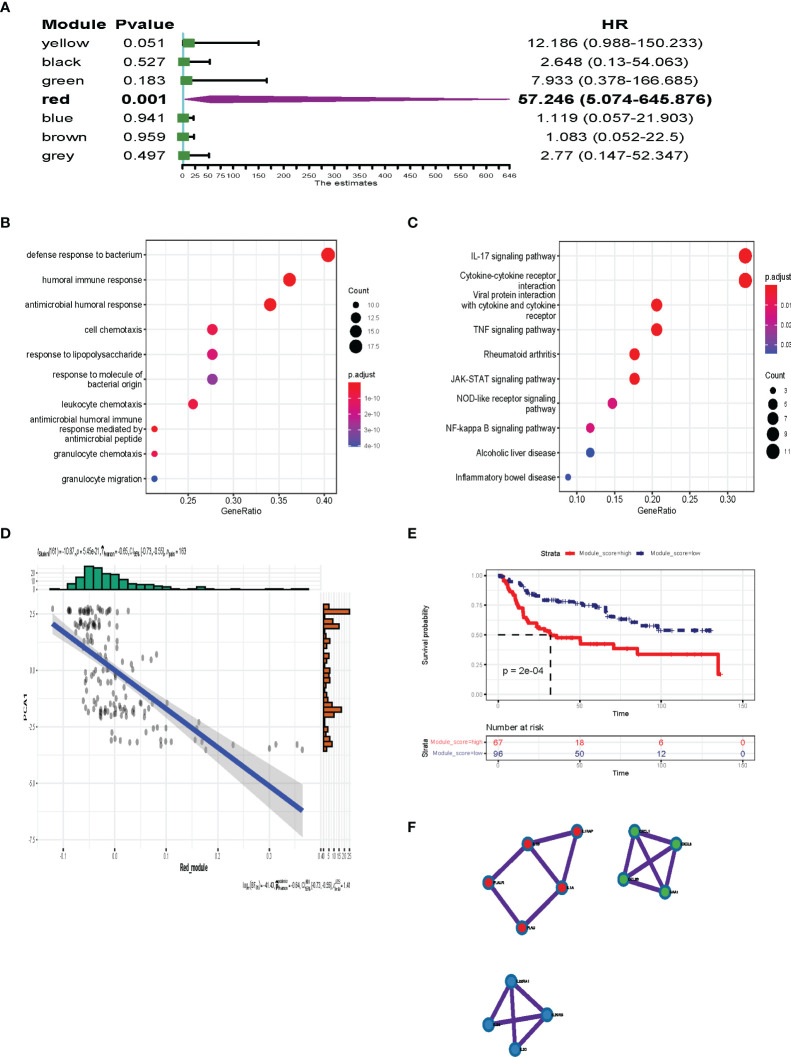
Identification of the hub gene for BLCA mRNA vaccines. **(A)** Forest maps of single factor survival analysis of 7 modules of BLCA. **(B, C)** Dot plot showing top 10 GO terms **(B)** and KEGG **(C)** in the red module. The dot size and color intensity represent the gene count and enrichment level, respectively. **(D)** Correlation between the red module feature vector and first principal component in the immune landscape. **(E)** Differential prognosis in the red module with high and low mean. **(F)** 13 hub genes identified in red module.

Survival analysis was performed to identify the most important gene modules. As shown in **Figure 10A**, the red gene module was highly associated with the prognosis of BLCA patients (*P* = 0.001, HR = 57.246). The red module genes are shown in **Table S3**. We extracted the red module genes and then performed GO and KEGG analysis. GO analysis showed that the red module was highly enriched in immune-related biological processes, including humoral immune response, antimicrobial humoral response, and leukocyte chemotaxis (**Figure 10B**). Additionally, signaling pathway analysis suggested that the red module was enriched in immune-associated and oncogenic pathways, including the IL-17 signaling pathway, TNF signaling pathway, JAK-STAT signaling pathway, and NF-Kappa B signaling pathway (**Figure 10C**). As shown in **Figure 10D**, there was a negative correlation between component 1 and the red module score (r = −0.65, *P* < 0.001), which indicated that red module genes may be biomarkers for patients who are not suitable for mRNA vaccines. The survival analysis showed a low level of red modules was associated with poor prognosis (**Figure 10E**). Protein–protein interaction analysis identified 13 hub genes including *IL1A*, *IL1B*, *IL1RAP*, *PLAU*, *PLAUR*, *IL22RA1*, *IL24*, *IL20*, *IL20RB*, *CXCL1*, *CCL20*, *CXCL6*, and *SAA1* (**Figure 10F**). These data show that the hub genes we identified could act as biomarkers for populations suitable for mRNA vaccines and predict the prognosis of patients.

## Discussion

BLCA, a highly heterogeneous cancer, is also one of the most aggressive and recurrent cancers (3, 26). With the development of sequencing technology, treatment strategies for advanced disease are undergoing rapid changes, as immunotherapy with checkpoint inhibitors, targeted therapies, and antibody-drug conjugates have become options for certain patients at various stages of disease (23). However, the effectiveness of these treatments for BLCA remains unknown. The mRNA tumor vaccine is an innovative immunotherapy that aims to elicit tumor-specific immune responses at a low-cost of manufacturing and high potency (27, 28). Research focused on the use of mRNA vaccines combined with other immunotherapies is underway (12, 29). However, limited studies exist on the development of anti-BLCA mRNA vaccines.

In this study, we filtered tumor-specific antigens by comprehensive analysis of the landscapes of mutational and amplified profiles in the TCGA-BLCA dataset. Somatic mutations in cancer cells can generate tumor-specific neoepitopes, which are recognized by autologous T cells in the host (30). Therefore, the antigens we detected could be attractive targets for therapeutic cancer vaccines. However, these candidate genes may not play a critical role in the immune microenvironment and prognosis. Subsequently, we integrated survival-related genes and performed immune infiltration analysis with APCs. Three of the identified candidate antigens (*AP2S1*, *P3H4*, *RAC3*) were strongly associated with inferior prognosis and had the potential to induce a significant immune response for identification by T cells. In summary, these genes were identified as candidate antigens for mRNA vaccine development, although further evaluation is required. Previous studies have confirmed the potential of these genes. Overexpression of *P3H4* indicated a poor prognosis in lung adenocarcinoma (31). In addition, *AP2S1* was confirmed as a risk factor and was correlated with immune infiltration in triple-negative breast cancer (32). *RAC3* was also demonstrated to promote invasion and metastasis in breast cancer and bladder cancer (33, 34).

With recent exploration of the tumor microenvironment, there is an increasing awareness of the impact of individual differences on cancer vaccine response (7, 25). Identification of specific vaccine-suitable populations is a key issue in mRNA vaccine development. The definitions of “hot” and “cold” tumors aid in predicting immunotherapy response, including response to mRNA vaccines (6). Therefore, to filter specific populations, the immune subtypes were classified into three types by immune-related profile. Both the TCGA and GSE13507 datasets showed consistent classification and prognosis. Low pathologic T stage and non-muscle invasive subtypes mapped with the BCS2 type, while those of BCS1 and BCS3 were very similar to each other. The poor prognosis of BCS1 and BCS3 might be caused by high pathologic T stage and invasiveness. Interestingly, the mutation landscape and TMB of the three types showed no significant differences. A previous study reported that the predictive value of a universal numerical threshold for TMB-high was limited, owing to variability across cancer types and unclear associations with survival outcomes (5). Therefore, screening suitable populations for mRNA vaccines based on TMB was not effective. Further exploration of the immune landscape was required. ICD plays a critical role in changing tumors from “cold” to “hot” and provides a rich source of immunogens for anti-tumor T cell cross-priming and sensitizing cancer cells to interventional immunotherapy (6, 25, 35). Our results showed that the BCS1 and BCS3 subtypes were associated with a high level of ICP, and the BCS2 subtype was associated with a high level of ICD. These results preliminarily suggested that BCS2 subtype patients might be a suitable mRNA vaccine population. A high level of ICP was correlated with suppression of the immune microenvironment, indicating the suitability of immune checkpoint inhibitors in BCS1 and BCS3 patients. Moreover, immune-related signature pathway analysis also demonstrated that signaling pathways such as chemokines were downregulated in the BCS2 subtype. The deficiency of specific chemokines may make it difficult for T cells to enter the interior of the tumor (36). Of note, the BCS2 subtype was also mapped with the C4 (lymphocyte depleted) type. The lymphocyte depleted subtype was correlated with a low fraction of lymphocytes and high fraction of macrophages (20). Immune infiltration analysis was performed and confirmed that the BCS2 subtype had low infiltration of lymphocytes. Therefore, low lymphocyte infiltration could be activated by mRNA vaccination. These results suggested that the previously identified immune subtypes and our immune subtypes consistently predicted lymphocyte fraction. In addition, novel immunophenotyping may complement the previous phenotyping.

The further immune landscape of dimensionality reduction revealed the heterogeneity within the BCS1 subtype, and the immune landscape showed that the positions of BCS1 and BCS3 were closely related to each other in the graph, indicating the accuracy of our model in distinguishing specific populations. More importantly, this was also consistent with the results of our previous staging prognostic analysis and suggests a possible prognostic indicator for BLCA. Ultimately, 13 hub genes were involved in immune-related signaling pathways (TNF signaling pathway, JAK-STAT signaling pathway, NF-kappa B signaling pathway). Previous studies reported that these pathways participate in the formation of the tumor microenvironment (36–38), especially regarding anti-tumor immunity and inflammation. Taken together, the data suggest that these hub genes may play a critical role in the tumor microenvironment.

In addition, our study provides important insight for mRNA vaccine development in cancer and other immune-related diseases. The application of mRNA vaccines combined with other immunotherapies may be a potential strategy to increase the likelihood of tumor cell eradication (29). Relevant clinical studies have been conducted and have initially confirmed the safety of this strategy (5, 39). However, our study also had some limitations. For example, the potential effect of the three antigens still requires exploration. In addition, the sample size of this study should be further expanded to identify and verify the accuracy and generalizability of the immune subtypes of BLCA.

## Conclusions

In conclusion, *AP2S1*, *P3H4*, and *RAC3* were identified as candidate tumor-specific antigens in BLCA, and BCS2 and BCS1A patients may benefit from mRNA vaccine therapy. Additionally, *IL1A*, *IL1B*, *IL1RAP*, *PLAU*, *PLAUR*, *IL22RA1*, *IL24*, *IL20*, *IL20RB*, *CXCL1*, *CCL20*, *CXCL6*, and *SAA1* were identified as suitable indicators for anti-BLCA mRNA vaccines. This study provides a novel insight for cancer mRNA vaccine development and application.

## Data Availability Statement

The original contributions presented in the study are included in the article/**Supplementary Material**. Further inquiries can be directed to the corresponding author.

## Author Contributions

GW and YG conceived the study, performed the literature search and bioinformatics analysis, and prepared the figures; YC, KW, and SZ helped with data collection, analysis, and interpretation. GW and YG wrote and revised the manuscript. The author(s) read and approved the final manuscript.

## Conflict of Interest

The authors declare that the research was conducted in the absence of any commercial or financial relationships that could be construed as a potential conflict of interest.

## Publisher’s Note

All claims expressed in this article are solely those of the authors and do not necessarily represent those of their affiliated organizations, or those of the publisher, the editors and the reviewers. Any product that may be evaluated in this article, or claim that may be made by its manufacturer, is not guaranteed or endorsed by the publisher.

## Abbreviations

BLCA, Bladder cancer; WGCNA, Weighted gene co-expression network analysis; TMB, Tumor mutation burden; OS, Overall survival; DFS, Disease-free survival; ICP, Immune check points; ICD, Immunogenic cell death; CNV, Copy number variation; SNV, Single nucleotide variation; TCGA, The Cancer Genome Atlas; ssGSEA, Single-sample GSEA.
