# Supplementary figures and images for: Identification of Novel Tumor Antigens and the Immune Landscapes of Bladder Cancer Patients for mRNA Vaccine Development
Source: Front Oncol. 2022 Jun 24;12:921711. doi: 10.3389/fonc.2022.921711 (PMC9263198; doi:10.3389/fonc.2022.921711)

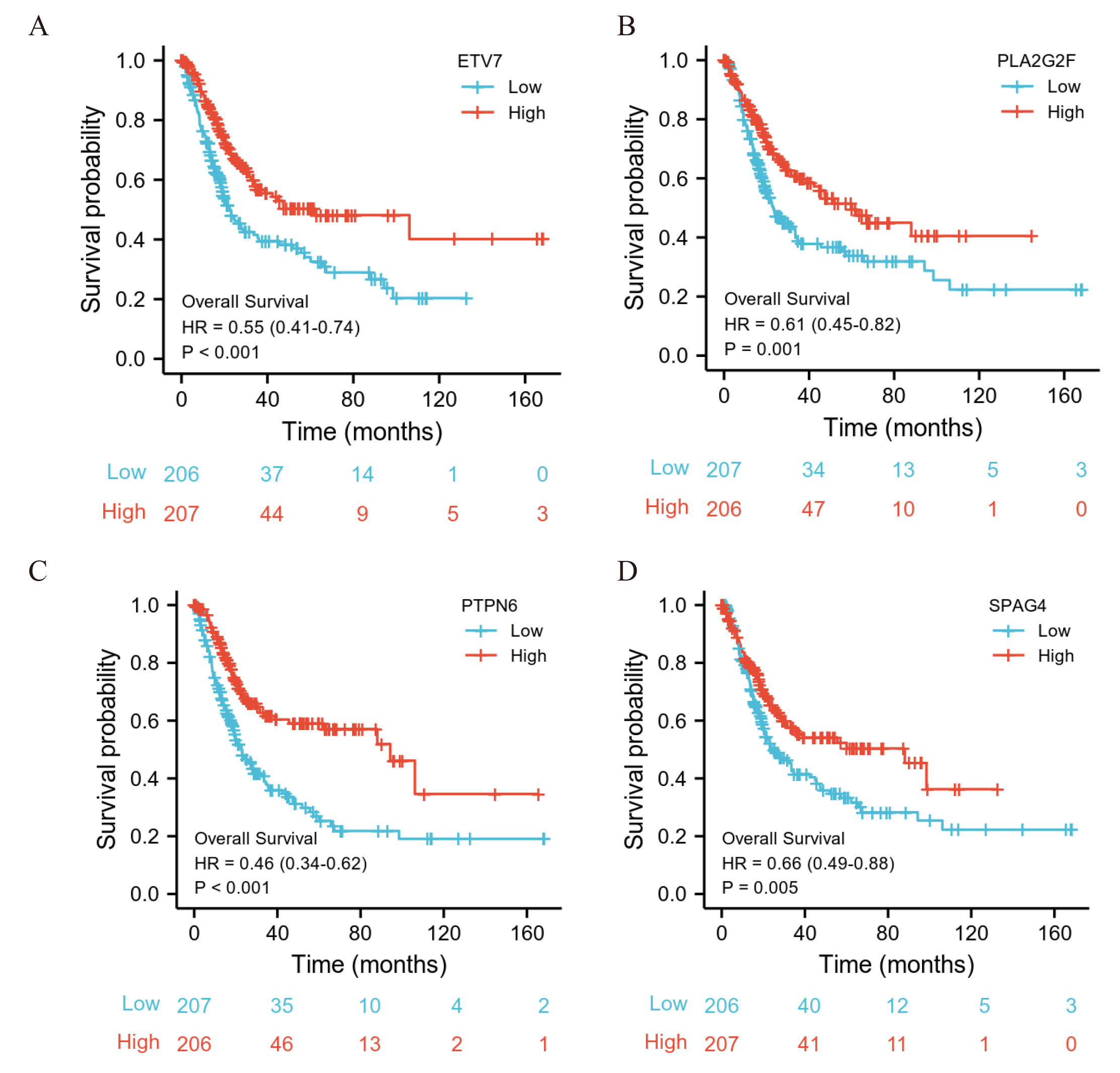

Supplement: Supplementary file 1 [file Image_1.tif]
